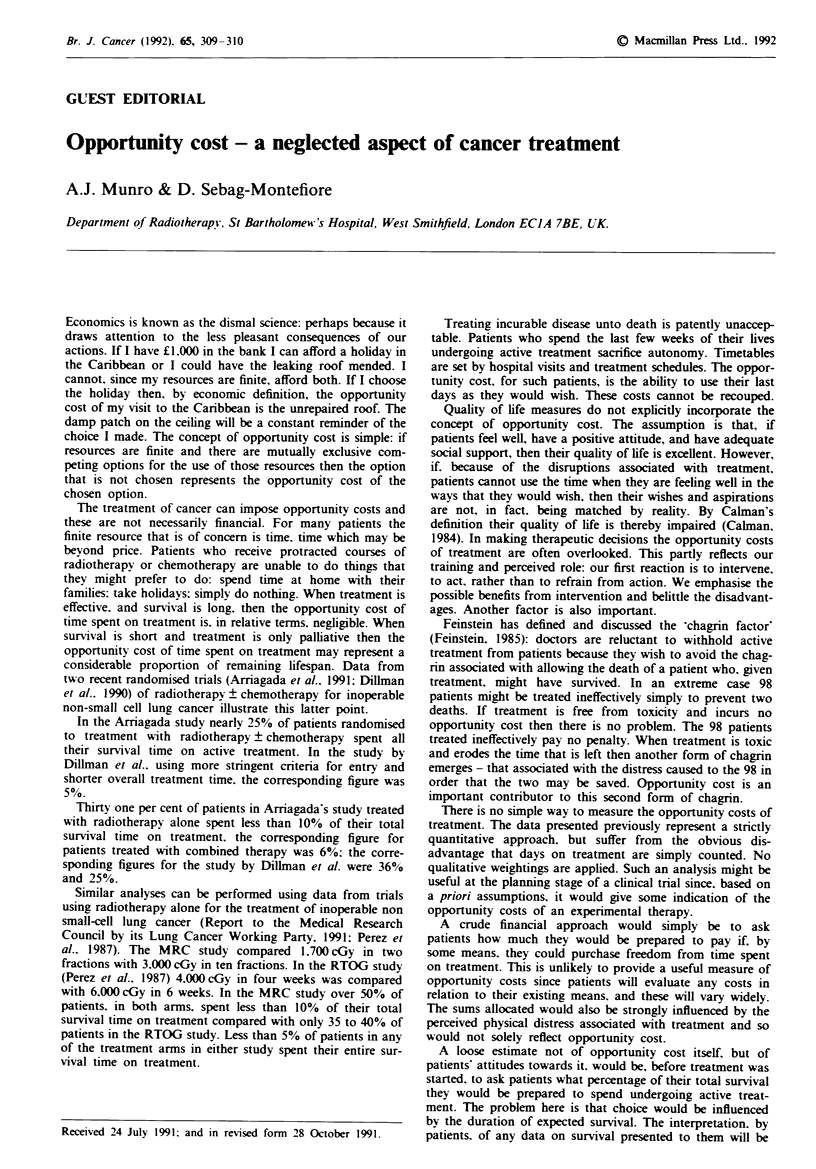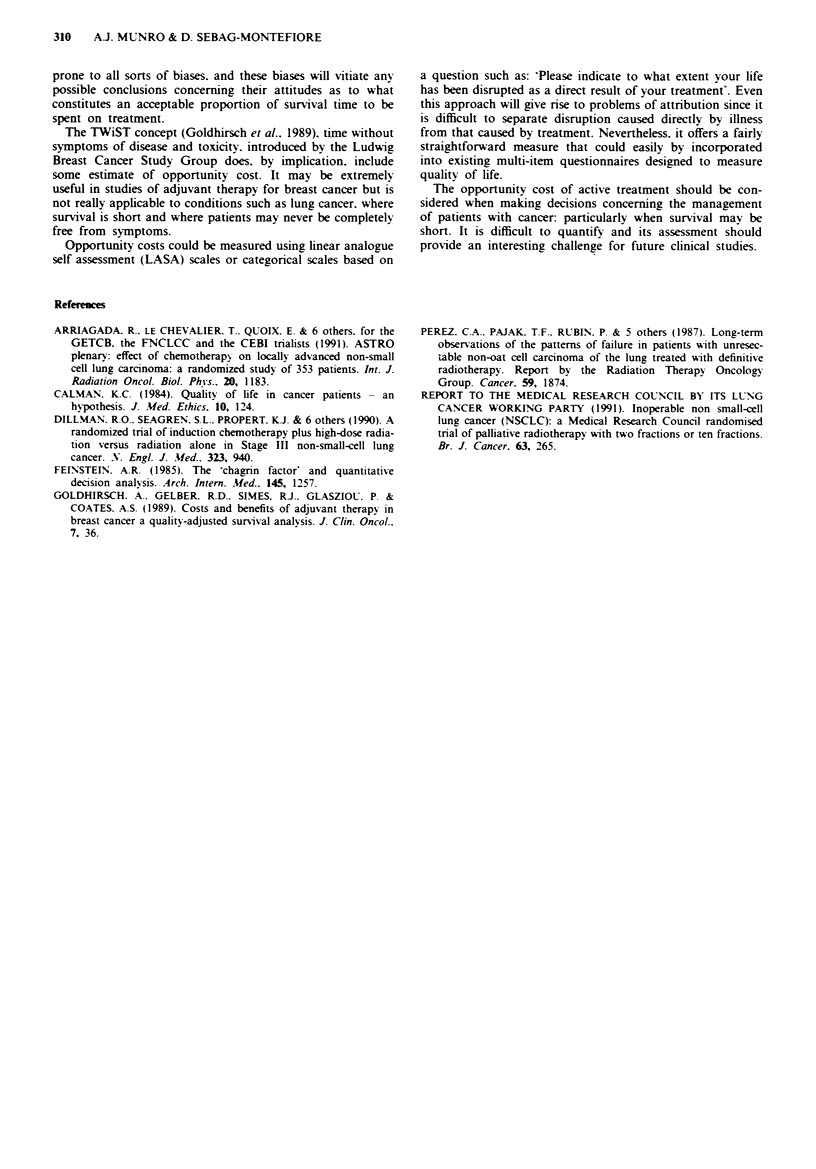# Opportunity cost--a neglected aspect of cancer treatment.

**DOI:** 10.1038/bjc.1992.64

**Published:** 1992-03

**Authors:** A. J. Munro, D. Sebag-Montefiore


Br. J. Cancer (1992), 65, 309-310 © Macmillan Press Ltd., 1992

GUEST EDITORIAL

### Opportunity cost – a neglected aspect of cancer treatment

A.J. Munro & D. Sebag-Montefiore

*Department of Radiotherapy, St Bartholomew's Hospital, West Smithfield, London EC1A 7BE, UK.*

Economics is known as the dismal science: perhaps because it draws attention to the less pleasant consequences of our actions. If I have £1,000 in the bank I can afford a holiday in the Caribbean or I could have the leaking roof mended. I cannot, since my resources are finite, afford both. If I choose the holiday then, by economic definition, the opportunity cost of my visit to the Caribbean is the unrepaired roof. The damp patch on the ceiling will be a constant reminder of the choice I made. The concept of opportunity cost is simple: if resources are finite and there are mutually exclusive competing options for the use of those resources then the option that is not chosen represents the opportunity cost of the chosen option.

The treatment of cancer can impose opportunity costs and these are not necessarily financial. For many patients the finite resource that is of concern is time, time which may be beyond price. Patients who receive protracted courses of radiotherapy or chemotherapy are unable to do things that they might prefer to do: spend time at home with their families; take holidays; simply do nothing. When treatment is effective, and survival is long, then the opportunity cost of time spent on treatment is, in relative terms, negligible. When survival is short and treatment is only palliative then the opportunity cost of time spent on treatment may represent a considerable proportion of remaining lifespan. Data from two recent randomised trials (Arriagada *et al.*, 1991; Dillman *et al.*, 1990) of radiotherapy ± chemotherapy for inoperable non-small cell lung cancer illustrate this latter point.

In the Arriagada study nearly 25% of patients randomised to treatment with radiotherapy ± chemotherapy spent all their survival time on active treatment. In the study by Dillman *et al.*, using more stringent criteria for entry and shorter overall treatment time, the corresponding figure was 5%.

Thirty one per cent of patients in Arriagada's study treated with radiotherapy alone spent less than 10% of their total survival time on treatment, the corresponding figure for patients treated with combined therapy was 6%; the corresponding figures for the study by Dillman *et al.* were 36% and 25%.

Similar analyses can be performed using data from trials using radiotherapy alone for the treatment of inoperable non small-cell lung cancer (Report to the Medical Research Council by its Lung Cancer Working Party, 1991; Perez *et al.*, 1987). The MRC study compared 1,700 cGy in two fractions with 3,000 cGy in ten fractions. In the RTOG study (Perez *et al.*, 1987) 4,000 cGy in four weeks was compared with 6,000 cGy in 6 weeks. In the MRC study over 50% of patients, in both arms, spent less than 10% of their total survival time on treatment compared with only 35 to 40% of patients in the RTOG study. Less than 5% of patients in any of the treatment arms in either study spent their entire survival time on treatment.

Received 24 July 1991; and in revised form 28 October 1991.

Treating incurable disease unto death is patently unacceptable. Patients who spend the last few weeks of their lives undergoing active treatment sacrifice autonomy. Timetables are set by hospital visits and treatment schedules. The opportunity cost, for such patients, is the ability to use their last days as they would wish. These costs cannot be recouped.

Quality of life measures do not explicitly incorporate the concept of opportunity cost. The assumption is that, if patients feel well, have a positive attitude, and have adequate social support, then their quality of life is excellent. However, if, because of the disruptions associated with treatment, patients cannot use the time when they are feeling well in the ways that they would wish, then their wishes and aspirations are not, in fact, being matched by reality. By Calman's definition their quality of life is thereby impaired (Calman, 1984). In making therapeutic decisions the opportunity costs of treatment are often overlooked. This partly reflects our training and perceived role: our first reaction is to intervene, to act, rather than to refrain from action. We emphasise the possible benefits from intervention and belittle the disadvantages. Another factor is also important.

Feinstein has defined and discussed the 'chagrin factor' (Feinstein, 1985): doctors are reluctant to withhold active treatment from patients because they wish to avoid the chagrin associated with allowing the death of a patient who, given treatment, might have survived. In an extreme case 98 patients might be treated ineffectively simply to prevent two deaths. If treatment is free from toxicity and incurs no opportunity cost then there is no problem. The 98 patients treated ineffectively pay no penalty. When treatment is toxic and erodes the time that is left then another form of chagrin emerges – that associated with the distress caused to the 98 in order that the two may be saved. Opportunity cost is an important contributor to this second form of chagrin.

There is no simple way to measure the opportunity costs of treatment. The data presented previously represent a strictly quantitative approach, but suffer from the obvious disadvantage that days on treatment are simply counted. No qualitative weightings are applied. Such an analysis might be useful at the planning stage of a clinical trial since, based on *a priori* assumptions, it would give some indication of the opportunity costs of an experimental therapy.

A crude financial approach would simply be to ask patients how much they would be prepared to pay if, by some means, they could purchase freedom from time spent on treatment. This is unlikely to provide a useful measure of opportunity costs since patients will evaluate any costs in relation to their existing means, and these will vary widely. The sums allocated would also be strongly influenced by the perceived physical distress associated with treatment and so would not solely reflect opportunity cost.

A loose estimate not of opportunity cost itself, but of patients' attitudes towards it, would be, before treatment was started, to ask patients what percentage of their total survival they would be prepared to spend undergoing active treatment. The problem here is that choice would be influenced by the duration of expected survival. The interpretation, by patients, of any data on survival presented to them will be prone to all sorts of biases, and these biases will vitiate any possible conclusions concerning their attitudes as to what constitutes an acceptable proportion of survival time to be spent on treatment.

The TWiST concept (Goldhirsch *et al.*, 1989), time without symptoms of disease and toxicity, introduced by the Ludwig Breast Cancer Study Group does, by implication, include some estimate of opportunity cost. It may be extremely useful in studies of adjuvant therapy for breast cancer but is not really applicable to conditions such as lung cancer, where survival is short and where patients may never be completely free from symptoms.

Opportunity costs could be measured using linear analogue self assessment (LASA) scales or categorical scales based on a question such as: 'Please indicate to what extent your life has been disrupted as a direct result of your treatment'. Even this approach will give rise to problems of attribution since it is difficult to separate disruption caused directly by illness from that caused by treatment. Nevertheless, it offers a fairly straightforward measure that could easily by incorporated into existing multi-item questionnaires designed to measure quality of life.

The opportunity cost of active treatment should be considered when making decisions concerning the management of patients with cancer: particularly when survival may be short. It is difficult to quantify and its assessment should provide an interesting challenge for future clinical studies.

### References

ARRIAGADA, R., LE CHEVALIER, T., QUOIX, E. & 6 others, for the GETCB, the FNCLCC and the CEBI trialists (1991). ASTRO plenary: effect of chemotherapy on locally advanced non-small cell lung carcinoma: a randomized study of 353 patients. *Int. J. Radiation Oncol. Biol. Phys.*, **20**, 1183.

CALMAN, K.C. (1984). Quality of life in cancer patients – an hypothesis. *J. Med. Ethics*, **10**, 124.

DILLMAN, R.O., SEAGREN, S.L., PROPERT, K.J. & 6 others (1990). A randomized trial of induction chemotherapy plus high-dose radiation versus radiation alone in Stage III non-small-cell lung cancer. *N. Engl. J. Med.*, **323**, 940.

FEINSTEIN, A.R. (1985). The 'chagrin factor' and quantitative decision analysis. *Arch. Intern. Med.*, **145**, 1257.

GOLDHIRSCH, A., GELBER, R.D., SIMES, R.J., GLASZIOU, P. & COATES, A.S. (1989). Costs and benefits of adjuvant therapy in breast cancer a quality-adjusted survival analysis. *J. Clin. Oncol.*, **7**, 36.

PEREZ, C.A., PAJAK, T.F., RUBIN, P. & 5 others (1987). Long-term observations of the patterns of failure in patients with unresectable non-oat cell carcinoma of the lung treated with definitive radiotherapy. Report by the Radiation Therapy Oncology Group. *Cancer*, **59**, 1874.

REPORT TO THE MEDICAL RESEARCH COUNCIL BY ITS LUNG CANCER WORKING PARTY (1991). Inoperable non small-cell lung cancer (NSCLC): a Medical Research Council randomised trial of palliative radiotherapy with two fractions or ten fractions. *Br. J. Cancer*, **63**, 265.